# Secondary ion mass spectrometry, a powerful tool for revealing ink formulations and animal skins in medieval manuscripts

**DOI:** 10.1098/rsos.230059

**Published:** 2023-06-07

**Authors:** David Gravis, Nicolas Roy, Nicolas Ruffini-Ronzani, Laurent Houssiau, Alexandre Felten, Nikolay Tumanov, Olivier Deparis

**Affiliations:** ^1^ Namur Institute of Structured Matter (NISM), University of Namur, Namur 5000, Belgium; ^2^ Heritages, transmissions, inheritances institute (PaTHs), University of Namur, Namur 5000, Belgium; ^3^ Namur Institute for Complex Systems (naXys), University of Namur, Namur 5000, Belgium; ^4^ Centre de recherche Pratiques médiévales de l’écrit (PraME), Department of History, University of Namur, Namur 5000, Belgium; ^5^ Synthesis, irradiation & analysis of materials (SIAM) technological platform, University of Namur, Namur 5000, Belgium; ^6^ Physico-chemical characterization (PC2) technological platform, University of Namur, Namur 5000, Belgium

**Keywords:** time-of-flight secondary ion mass spectrometry, cultural heritage, surface analysis, X-ray diffraction, attenuated total reflectance Fourier transform infrared spectroscopy, parchment and inks

## Abstract

Book production by medieval scriptoria have gained growing interest in recent studies. In this context, identifying ink compositions and parchment animal species from illuminated manuscripts is of great importance. Here, we introduce time-of-flight secondary ion mass spectrometry (ToF-SIMS) as a non-invasive tool to identify both inks and animal skins in manuscripts, at the same time. For this purpose, both positive and negative ion spectra in inked and non-inked areas were recorded. Chemical compositions of pigments (decoration) or black inks (text) were determined by searching for characteristic ion mass peaks. Animal skins were identified by data processing of raw ToF-SIMS spectra using principal component analysis (PCA). In illuminated manuscripts from the fifteenth to sixteenth century, malachite (green), azurite (blue), cinnabar (red) inorganic pigments, as well as iron-gall black ink, were identified. Carbon black and indigo (blue) organic pigments were also identified. Animal skins were identified in modern parchments of known animal species by a two-step PCA procedure. We believe the proposed method will find extensive application in material studies of medieval manuscripts, as it is non-invasive, highly sensitive and able to identify both inks and animal skins at the same time, even from traces of pigments and tiny scanned areas.

## 1. Introduction

Medieval illuminated manuscripts written on parchment are an important and valuable heritage of the world culture. Over centuries, the almost exclusive writing support in medieval Europe was parchment, a complex biological material processed from animal skin, mainly calf, sheep and goat [1–3]. Inks, not only used for writing but also for decorating, were prepared thanks to sophisticated mixtures of organic or inorganic compounds in solution [4–6]. In studies of medieval manuscripts, scholars are increasingly concerned by the materiality of writing [1]. In codicology, a discipline that studies the materiality of manuscript books, there is a growing interest in questions related to the production of manuscripts and the economy of parchment [7–9] and inks [10]. Analytical techniques, when applied to the characterization of parchments and inks, are expected to contribute to the elucidation of such questions while enriching our historical knowledge about manuscripts.

A wide range of analytical techniques is currently involved in the material study of manuscripts. As far as inks are concerned, X-ray fluorescence (XRF) is commonly used to quantify the elemental composition of inks such as iron-gall inks (black inks used for texts) [11]. This elemental technique has the advantage of being able to analyse large parchment areas at once and is non-destructive. Yet, XRF can be challenging for the identification of organic-based inks. Complementary to XRF, X-Ray diffraction (XRD) can be used to analyse and semi-quantify the crystalline phases of ink pigments [12]. Even though powder XRD requires invasive sampling of ink layers in some configurations, it is possible to adapt it to non-destructive analysis [13]. However, XRD is not suited for analysis of non-crystalline or organic compounds. To characterize amorphous and organic inks, techniques such as attenuated total reflection Fourier transform infrared (ATR-FTIR) [14,15] and Raman [16] spectroscopies are available. The fact that some inorganic pigments are transparent in mid-IR [17] prevents the use of ATR-FTIR in these case, however. As far as parchments are concerned, various analytical techniques have long been used to assess their level of conservation in museum and library collections [18]. Artificial ageing [19] experiments on modern parchments have also been conducted in order to predict parchment degradation due to various environmental factors and help optimize conservation conditions as well as restoration treatments. More recently, techniques have been introduced in order to identify animal species in parchments [20–22]. Thanks to non-destructive triboelectric sampling of the parchment surface, non-invasive proteomic mass fingerprint analysis of parchment collagen (also called ZooMS for zooarchaeological mass spectrometry) has been increasingly used to determine animal species in medieval manuscripts [2,7,23]. Non-invasive identification of parchment animal species was reported as well, using spectrophotometry combined with principal component analysis (PCA) [22]. PCA was also applied to time-of-flight secondary ion mass spectrometry (ToF-SIMS) data for parchment species recognition [24].

So far, to our knowledge, ToF-SIMS has been scarcely used for characterization of parchments [24,25] and inks [26–30]. ToF-SIMS was used successfully to identify pigments in other works of art, such as blue copper-based pigments in paintings—with further quantification of the paint degradation [26], azurite and other paint components (animal glue for instance) [27], lead white and cinnabar [29], gilding and silver, as well as green, blue and white organic or inorganic pigments on leather substrates [28]. Nevertheless, when fragments were not available, these studies required invasive sampling for cross-section analysis. A methodology relying on plastic substrate impregnated with chelators was reported [30] to capture metallic particles from artefact surfaces, reducing the invasiveness of the sampling. Metallic particles were then identified by mass spectrometry. While resolving the invasive sampling issue, this technique is limited to the identification of mineral pigments. Whether or not ToF-SIMS could be regarded as non-destructive in the context of manuscript studies is questionable though. If ToF-SIMS could be applied safely on inked parchments, it would become an interesting alternative. Indeed, by moving from inked to non-inked areas of the parchment, recording ion mass spectra and processing data, one could obtain information on ink composition and parchment animal species at the same time, which offers a potentially more complete characterization of manuscripts using a single technique.

**Figure 1.**
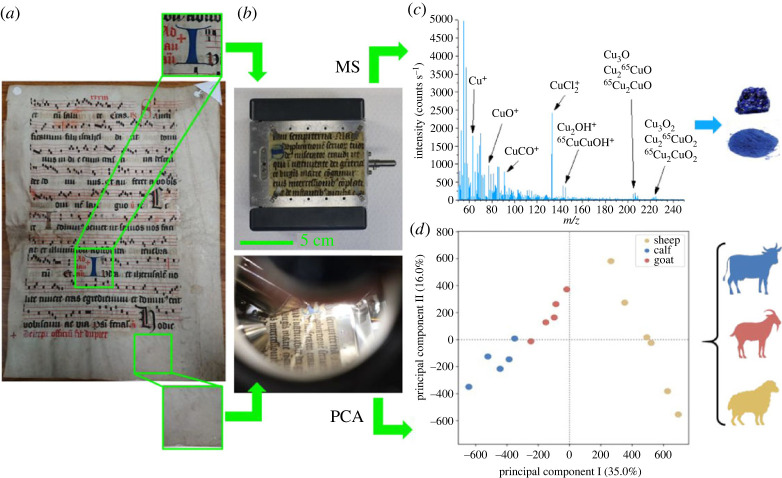
Methodology of ToF-SIMS analysis of illuminated manuscripts. (*a*) Example of manuscript (estimated from fifteenth to sixteenth century). Both inked areas (blue dropped initial ‘I’) and ink-free areas were analysed by ToF-SIMS; (*b*) mass spectra (MS) were directly analysed in order to identify ink pigments while PCA was performed on multiple MS obtained from the parchment bare surface, in order to discriminate animal species. (*c*) Secondary ion MS in positive polarity for analysis of the blue pigment used in the dropped initial ‘I’. (*d*) PCA applied to negative polarity MS from parchments of different animal species (calf, sheep and goat). Data were taken on both sides of the skin (grain–flesh), in the same region of the skin, near the belly.

In this article, we first show that ToF-SIMS can be regarded as a safe characterization technique in the context of manuscript material studies. Then, we introduce ToF-SIMS as a novel non-invasive technique for ink and animal skin identification in illuminated manuscripts. For a proof of principle, inked or non-inked areas of tens of historical parchments (figure 1*a*) were analysed by ToF-SIMS (figure 1*b*). In inked areas, such as dropped initials, recorded mass spectra offered readily available information on ink chemical composition (figure 1*c*). Knowing typical chemical compositions of inks used to write or decorate manuscripts, their direct identification was achieved by searching for characteristic mass peaks of the ink's main compounds or ion fragments. Identification of animal species in modern parchment was achieved by applying PCA twice, first on raw MS data to remove intraclass variance then on the reduced dataset. Even without this two-step procedure, animal species identification was possible by applying PCA on raw MS spectra taken in the same region of the skin only (figure 1*d*).

## 2. Results

### 2.1. Time-of-flight secondary ion mass spectrometry analysis is safe for manuscripts

Our aim is to analyse both ink and parchment materials in illuminated manuscripts using the same technique. A preliminary requirement to be checked before applying TOF-SIMS to such invaluable cultural heritage objects is that the analysis leaves the manuscript unaltered afterwards. For this purpose, a sample was cut from a sacrificial manuscript in order to check if a typical ToF-SIMS session does not alter the sample both visually and at material microscopic level (see electronic supplementary material). The sample size (9 × 5 cm^2^) was the largest one afforded by the sample holder inside the ToF-SIMS instrument. In libraries or museum collections, however, manuscripts often take the form of codices, i.e. bound books with folded parchment leaves, and their sizes are much larger. Moreover, single folios of small size (comparable to the size of our sacrificial sample) are rather uncommon, except, for instance, in charters. The issue of the sample size limitation will be discussed at the end of this section.

On the material level, we proved that ToF-SIMS is not invasive *per se*. In fact, after a typical ToF-SIMS session which included a long period of depressurization, neither degradation of the visual aspect of the manuscript nor significant change in its dimensions could be observed (see electronic supplementary material, figure S2), which is a fully valid argument for conservation purpose. The sputtering of material by the ion probe beam during analysis only concerns the very first atomic layers of the surface (i.e. a few nanometres in depth). Moreover, a quick scan of the surface is enough so that no erosion of the surface is needed to obtain the key information from emitted secondary ions. Such an extremely low level of material removal from the surface is already accepted for other analytical techniques used in manuscript material studies. For instance, in ZooMS studies [31], parchment sampling is considered as non-invasive in spite of the fact it consists of triboelectric extraction of a tiny amount of parchment's collagen through gentle rubbing of the parchment surface with PVC-free eraser, an operation which is also commonly done for cleaning parchment surfaces [32]. Beyond visual aspect and macroscopic dimensions, we also showed that ToF-SIMS did not alter the parchment on the microscopic level (see electronic supplementary material, figure S2). Indeed, long exposure to vacuum during a typical ToF-SIMS session had no effect on the collagen structure of the parchment, as checked by ATR-FTIR. Actually, exposure to vacuum can even be used for recovery of damaged parchments (for instance, due to microbial degradation) [33]. Only a slight loss in moisture could be observed, but the parchment, which is known to be quite resilient to hydrothermal stress, was able to quickly equilibrate itself after exposure back to the atmosphere. We can conclude that historical manuscripts can safely withstand the relatively long period of depressurization during ToF-SIMS analysis.

The last remaining issue with ToF-SIMS analysis of manuscripts is the invasiveness regarding the object itself. Commercially available ToF-SIMS instruments can analyse samples of maximum size of 100 cm^2^ (example of sample in electronic supplementary material, figure S2). While small manuscript fragments exist (Dead Sea scrolls pieces for instance), solutions must be found to adapt the technique to avoid cutting large pieces in precious manuscripts. For example, it has been shown that microscopic removal of material from the surface is acceptable and sufficient for medieval painting studies with ToF-SIMS [34,35]. Similarly, with due authorizations, it could be allowed to remove tiny parchment pieces from hidden regions e.g. near binding or in already damaged areas. If such sampling is not permitted, the limiting factor is the size of the vacuum chamber and the pumping system's efficiency. A bigger analysis chamber, as well as a pre-vacuum chamber and transfer system of comparable sizes, are thus needed for applying the technique to the study of manuscripts from collections. If this upscaling is still a technical and economic issue, new ToF-SIMS techniques—MeV-SIMS [36,37]—can operate at atmospheric pressure. These instruments, however, operate with primary ions of high kinetic energy (several MeV), and their interaction with organic materials has not been fully studied yet.

### 2.2. Identification of inks by time-of-flight secondary ion mass spectrometry

**Figure 2.**
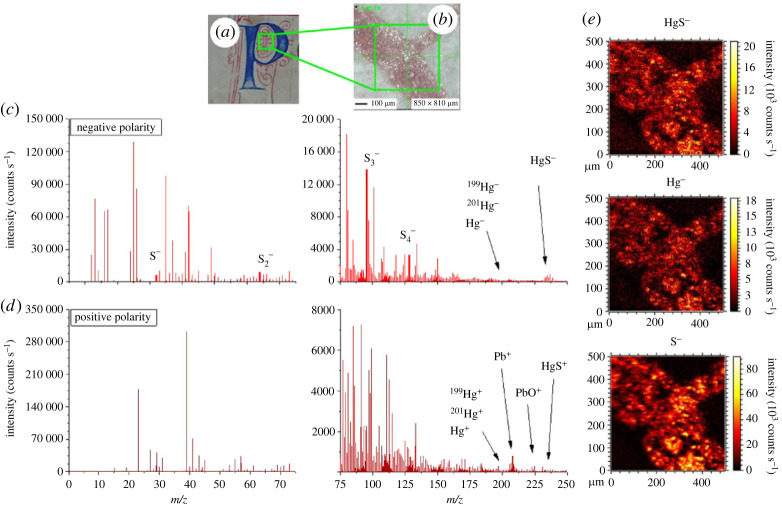
ToF-SIMS analysis of red pigment in both positive and negative polarities. (*a*) Photograph of the inked area; the (red) pigment was used for decoration around the blue *‘P’* dropped initial. (*b*) Microscope image of the inked area analysed by ToF-SIMS taken when the sample was in the analysis chamber. ToF-SIMS spectra in negative (*c*) and positive (*d*) polarities. Both spectra were cut off for clarity by discarding high masses (250–860 *m/z* not shown). (*e*) Mapping of characteristic negative ions associated with the red ink; from top to bottom: HgS**^−^**, Hg**^−^** and S**^−^**.

Hereafter, we demonstrate through a few examples that ToF-SIMS is a reliable technique for identification of pigments. ToF-SIMS was first used for analysis of red ink from a letter decoration (figure 2*a*), of which the microscope image taken with the sample inside the instrument (figure 2*b*), and the SIMS spectra (figure 2*c,d*, respectively, negative and positive polarities) are shown. High-intensity peaks, especially below 45 *m/z*, indicate a strong contribution of organic compounds. These organic molecules, which have limited interest for ink identification, also possess peaks at higher masses and could originate from either organic component entering into the ink formulation (fillers, viscosity agents, stabilizers, etc.) or dirt contamination on the inked surface (cleaning action in sample inked areas had to be as soft as possible). Pigment identification is, however, possible by examining other characteristic peaks. Mercury species, as well as associated atomic ions, present low sensitivity in both polarities. Nevertheless, elemental mercury (^200^Hg^−^), two of its main isotopes (^199^Hg^−^ and ^201^Hg^−^) and the double-ionized atom (^200^Hg^2−^) are detected in the negative polarity spectrum (figure 2*b*). Mercury sulfide species are also observed in the form of HgS^−^, but the low intensity of this anion makes it difficult to show up at higher masses (*m/z* > 250). Sulfur species signals are more intense, so that elemental sulfur ions (S^−^), as well as sulfur clusters ($S_{x}^{-},\;\mathrm{with}\;\;1\leq x\leq 6)$, can be easily identified. Presence of mercury sulfide-like ions, as well as corresponding elemental ions, indicates that the red pigment is cinnabar (mercury sulfide: HgS), a pigment commonly found in illuminated manuscripts for bright red colours [17,38]. Spectral mapping of the main species (figure 2*e*) show excellent superimposition with the inked area of the microscope image (figure 2*b*). Likewise, other higher *m/z* species (heavier sulfur clusters and ${\mathrm{HgS}}_{x}^{-}$) can be identified in the inked area (see electronic supplementary material, figure S4). Although mercury and sulfur species have lower sensitivities in positive polarity (figure 2*d*), all the aforementioned anions have their respective counterpart cations detected. Indeed, ^200^Hg^+^, ^199^Hg^+^, ^201^Hg^+^, ^200^Hg^+^, HgS^+^ and $S_{x}^{+}$ peaks are detected, further asserting the identification of cinnabar. Cerussite (lead carbonate: PbCO_3_ often accompanied with its hydrated form hydrocerussite 2[PbCO_3_]·Pb(OH)_2_) can be detected too, especially in positive polarity (figure 2*d*), as mainly lead (Pb^+^) and lead oxides (PbO^+^) fragments can be observed with strong intensity. Cerussite, a white mineral, has been often used on purpose by ink manufacturers in order to endow specific visual characteristics to the ink, for example giving shimmering effect or brighter hues. The difference in intensity between lead and mercury species is only due to species-dependent sensitivity that is inherent to the technique (i.e. mostly due to different sputtering and ionization yields of each species). For this reason, such an analysis cannot give quantitative information on the ink composition based on the relative intensities of the peaks. In summary, we can conclude that the analysed red pigment is a mixture of cinnabar and cerussite.

**Figure 3.**
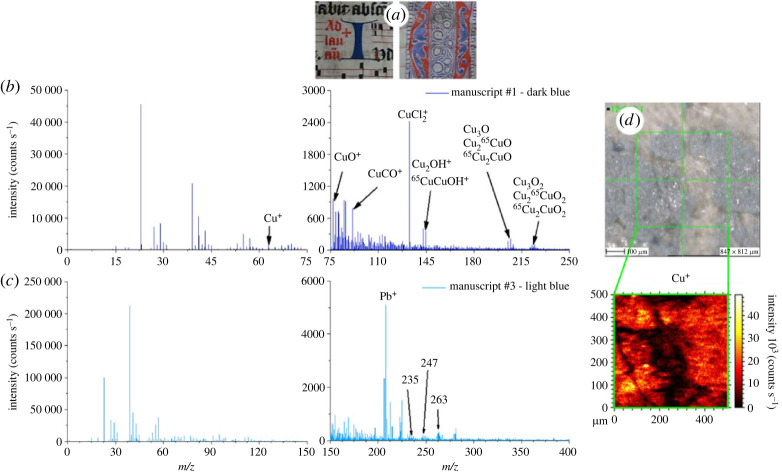
ToF-SIMS analysis of two different blue pigments. (*a*) Photographs in inserts show two analysed inked areas; dark blue dropped initial ‘I’, light blue decoration of the ‘O’ dropped initial. ToF-SIMS spectra (*b*,*c*) in positive polarity of dark blue (*b*) and light blue (*c*) pigments. (*d*) Microscope image of the dark-blue inked area taken when the sample was in the analysis chamber and corresponding mapping of the characteristic Cu^+^ ions.

Similarly, two types of blue inks, dark blue and light blue, were analysed (figure 3*a*). In these cases, only positive polarity spectra are shown (figure 3*b,c*) because the analysed inks exhibit better sensitivity in this polarity. In general, when no *a priori* knowledge is available about the pigment, both polarities should, however, be considered. For the dark blue ink, the identified pigment is azurite (Cu_3_(CO_3_)_2_(OH)_2_), which contains copper, an element more easily detected in positive polarity as Cu^+^ (figure 3*b*). ^63^Cu^+^ ion is observed too, yet with a rather low intensity (around 2000 counts/s). Other copper compounds are better identified, with high intensities considering their multi-elements nature (from 250 to 1750 counts/s). More specifically, fragments of copper oxides (CuO^+^, Cu_3_O^+^, ${\mathrm{Cu}}_{3}O_{2}^{+}$), copper hydroxides (Cu_2_OH^+^) and copper carbonates (CuCO^+^) are identified. These assignments are confirmed by isotopic distribution for peaks that correspond to species containing the ^65^Cu radioisotope (not highlighted in figure 3*b* for clarity). Other low-intensity peaks of copper species are also identified at higher masses, such as ${\mathrm{Cu}}_{3}H_{2}O_{2}^{+}$, CuSO^+^, CuCO_2_ and ${\mathrm{Cu}}_{4}O_{2}^{+}$ with their corresponding isotopes. It should be noted that ${\mathrm{CuCl}}_{2}^{+}$ is identified as well, which could result from the sputtering of fragments of copper chloride, the latter originating from copper-chlorine reaction products. Chlorine, as contaminant, is certainly present on the parchment surface due to its exposition to environment. Indeed, the most probable sources of chlorine being salts such as of alkaline metals, on account of the identification of such elements (sodium, potassium). They could be present in water used during parchment fabrication, or simply naturally present inside the animal skin. Traces of zinc (^64^Zn^+^), confirmed by the presence of ZnOH^+^, are detected. Zinc crystalline compounds are often observed in azurite and could explain why it is detected here. However, their low peak intensities suggest a low concentration in the natural azurite mineral used for ink preparation. The identification of copper and its compounds (mainly carbonates and hydroxides) shows that ToF-SIMS is quite sensitive for the detection of azurite. Once again perfect superimposition of the inked area observed by microscopy with the ion mapping of the pigment is achieved (figure 3*d*).

The light blue ink does not show the typical azurite mass peaks, while more organic peaks are detected both at low and high masses (figure 3*c*). This ink is thus certainly organic, probably indigo (C_16_H_10_N_2_O_2_), well known as blue pigment used in manuscripts. Therefore, we focus on the 150–300 *m/z* range which is richer in information for such an organic material. The identification is more challenging because other organic-based materials used in the ink formulation interfere in that range. Yet, three of the main indigo peaks [39] (at 263, 247 and 235 *m/z*) can be identified with relatively low deviation in peak positions (less than 0.01%), but also with rather low intensities, for example 370 counts s^−1^ for the main molecular ion C_16_H_10_N_2_O_2_^+^ (at 263 *m/z*). The two other main peaks correspond to fragments, i.e. C_16_H_10_N_2_O^+^ (at 247 *m/z*) and C_15_H_10_N_2_O^+^ (at 235 *m/z*). The low intensity of the indigo characteristic peaks indicates a low sputter yield and certainly a low concentration in the ink mixture, which could explain the lighter blue coloration of the ink in regard to pure indigo. This hypothesis is supported by comparison with the intensities of 208 and 224 *m/z* peaks, which correspond to, respectively, Pb^+^ and PbO^+^, typical of cerussite used as filler. The rather low concentration of indigo makes it difficult to be detected by ToF-SIMS or any other analytical method, as the expected contributions of the other ion fragments related to indigo are even less intense and most likely hidden among the contributions of ink's components. Nevertheless, the absence of mass peaks from other blue ink candidates allows us to infer it is most likely indigo.

Other inks were analysed by ToF-SIMS and identified by searching for characteristic peaks of constitutive elements and molecular fragments of the pigments: the identification of black iron-gall ink (iron sulfate) in written text, gilding (gold), yellow ink and green (malachite) in decoration, can be found in the (electronic supplementary material, figure S5). Successful identification was achieved in all cases, in spite of the small amount of ink on the parchment surface. As for red and blue inks, perfect match between the inked areas (microscope images) and the spectral mapping of the elements was obtained.

### 2.3. Validation by other analytical techniques

**Figure 4.**
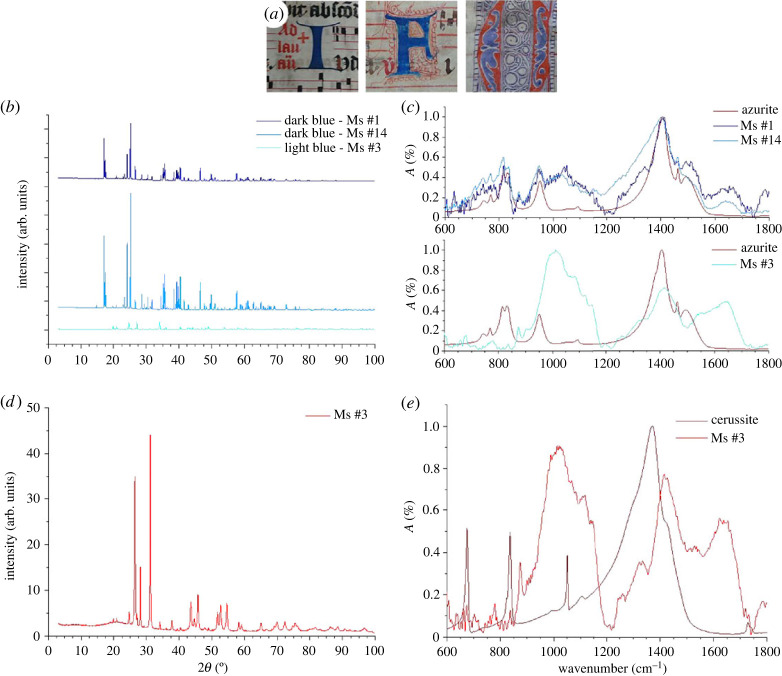
Identification of ink pigments in illuminated manuscripts performed with complementary analytical techniques. (*a*) Photographs of the analysed inked areas; from left to right: dark blue inks: blue ‘I’ and ‘F’ dropped initials, light blue decoration of the ‘O’ dropped initial; red ink: red part of the ‘O’ dropped initial. Powder-XRD (*b*,*d*) and ATR-FTIR spectra (*c*,*e*) of blue (*b*,*c*) and red (*d*,*e*) inks. ATR-FTIR reference spectra of the identified minerals are also shown.

In order to further validate the results obtained by ToF-SIMS, we resorted to two other analytical techniques commonly used for the identification of ink pigments, namely XRD and ATR-FTIR. For blue inks, three parchments (figure 4*a*) were used for either destructive (XRD) or non-invasive (ATR-FTIR) counter analyses. The ink was scraped from the parchment and analysed by powder XRD (figure 4*b*). The XRD spectra of dark blue inks used in two manuscripts (#1 and #14) are very similar, sharing numerous features, while the XRD spectrum of light blue ink used in another manuscript (#3) has very few and low-intensity peaks. Comparison with database indicates that the ink from manuscripts #1 and #14 is composed of around 90% of azurite and 10% of quartz. Quartz could be a natural inclusion inside the mineral or used as a filler in order to alter the colour of azurite pigment. These results validate our previous identification by ToF-SIMS. Contrary to ToF-SIMS, no zinc crystal is detected by XRD in blue inks from parchments decorated with azurite, most likely because its amount in the ink sample is too low. Presence of cerussite (lead carbonate) in light blue ink on parchment #3 is confirmed. In this sample, cerussite is the only crystalline structure to be detected, indicating the light blue pigment is either amorphous or organic, which is consistent with suspected indigo. Although XRD allows us to quantify the ink composition, it is a destructive method (scrapping of ink areas) which cannot be employed in the context of manuscript conservation.

ATR-FTIR spectra were also recorded (figure 4*c*) for all inks directly on the manuscripts. In the FTIR spectra of parchments #1 and #14, the main bands of azurite (reference spectrum) are present: bands at 1460, 1405, 950, 930, 815, 770 and 740 cm^−1^ are clearly observed whereas two minor bands at 1500 and 1090 cm^−1^ cannot be found, most likely hidden by contributions from other ink components. Nevertheless, in both manuscripts, a good correlation is obtained between the dark blue pigments and the reference spectrum of azurite. The presence of noise, as well as other bands, could be explained either by poor contact of the sample with the ATR crystal (reducing the intensity and resolution), or by the other components of the ink, such as fillers or organic compounds. It is important to note that quartz, an ink component in both manuscripts #1 and #14, could not be detected here, because (i) its concentration in the ink is low, and (ii) quartz has almost no active bands in the infrared range except for a low-intensity band at 870 cm^−1^, which could not be observed in the recorded spectra. Similarly, zinc compound is not detected again because the technique is not sensitive enough to detect such small traces.

The ATR-FTIR spectrum of the light blue ink (figure 4*c*) exhibits strong signals in the infrared range. Yet, the observed bands are rather broad, and unequivocal interpretation is risky due to the suspected low amount of blue pigment in the ink formulation, as already pointed out above. By comparison with the reference cerussite spectrum, which consists of four main bands, two of them can be identified (at 677 and 837 cm^−1^), while the main cerussite broad band at 1370 cm^−1^ could contribute to the shoulder of the band observed around 1420 cm^−1^. The two cerussite bands at 1057 and 1106 cm^−1^ are, however, not observed. By careful examination of the ATR-FTIR spectrum, our previous identification of indigo could be confirmed. Indeed, the broad band from 1550 to 1640 cm^−1^ could be characteristic of the aromatic ring of indigo, which involves coupling with respectively C=C and C=O vibrations (stretching). C=O rocking and wagging could be characteristic of the shoulder at 1060 cm^−1^ and the band at 680 cm^−1^, respectively. C-H and N-H stretching, bending and rocking can also be included within the wide bands centred around 1014 and 1431 cm^−1^, but the low resolution of the instrument (4 cm^−1^) does not allow us to separate each contribution. In the same spectral range, six-membered rings stretching and bending, as well as ring breathing could occur. A broad band at 3285 cm^−1^ (not shown in the spectrum) could also be characteristic of N-H stretching. All these features strongly suggest the presence of indigo in the light blue ink.

For red ink (figure 4*a*), the XRD pattern (figure 4*d*), when it is compared with the database, indicates that the crystalline phase of the red pigment on manuscript #3 contains 90% of mercury sulfide (cinnabar) and 10% of lead carbonate (cerussite). This result again validates our previous identification by ToF-SIMS. The ATR-FTIR spectrum (figure 4*e*) was also recorded. Cerussite can be identified again. Three of the four main cerussite bands (reference spectrum) can be observed: the bands at 677 and 837 cm^−1^ and the main broad band at 1370 cm^−1^. The cerussite band at 1050 cm^−1^ is, however, difficult to observe, probably because it is hidden in a broad-band contribution from the other components of the ink sample ranging from 950 to 1200 cm^−1^. Again, all the broad peaks observed in the red ink spectrum could originate from other organic components or surface contaminations, making identification difficult. Unfortunately, cinnabar is transparent to infrared radiation. In consequence, ATR-FTIR does not allow us to fully identify the red ink pigment.

Our previous ToF-SIMS identifications of other pigments were successfully validated by XRD and ATR-FTIR. Some examples can be found in the supporting information: green malachite (electronic supplementary materia, figure S3a,b) is easily identified by both XRD and ATR-FTIR whereas carbon black ink (electronic supplementary material, figure S3c) is identified solely by XRD. Indeed, in manuscripts, black inks are either carbon black ink or iron-gall ink. No crystalline structure being detected by XRD, this indicates that the black ink does not contain metal sulfates and is thus certainly carbon-based.

### 2.4. Identification of animal skins by time-of-flight secondary ion mass spectrometry

**Figure 5.**
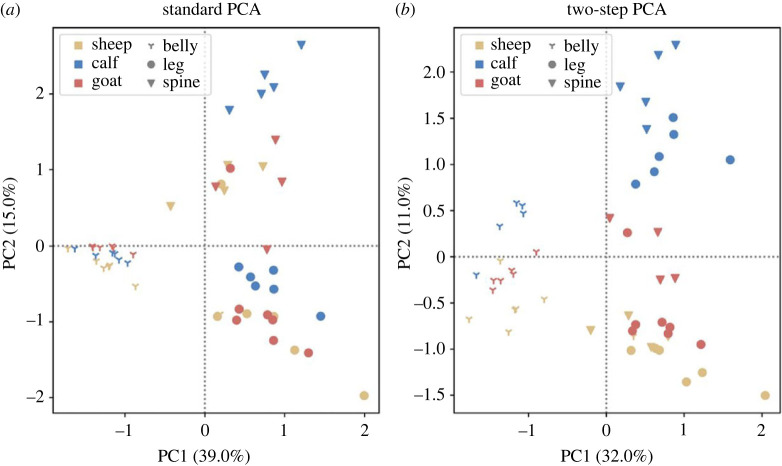
Identification of animal skins by ToF-SIMS with the help of PCA. Two-dimensional PCA (with corresponding variances to PC1 and PC2 in parenthesis) was applied to spectra recorded from the three animal species, on flesh side, in negative polarity. Three positions on the skins are considered: near the spine, the belly and the leg. (*a*) PCA performed on raw data, without intra-class variance removal. (*b*) Animal skin species identification of sheep, goat and calf with two-step PCA.

ToF-SIMS spectra in negative polarity were acquired from modern parchments (flesh side) manufactured from the three relevant animal species (goat, calf, sheep). PCA was directly applied to raw data (figure 5*a*), i.e. *m/z* intensity channels. While automatic peak shift (mass) calibration and time-of-flight correction were applied using the instrument software, no peak attribution was performed. This means that every single *m/z* channel was considered in the PCA procedure. One could argue that the physico-chemical information carried by each ionic species mass peak (with finite full width at half maximum (FWHM)) is overlooked, as the analysis was only performed at mathematical level. Yet, our procedure offers the advantage of avoiding any interpretation bias (either from the operator or the analysis software) as many small peaks could be hidden by broader and higher intensity peaks. Because they often appear as shoulders of higher intensity peaks, many ionic species are sometimes lost using automatic peak selection procedures. Refining our peak selection procedure and comparing results with automatic selection of finite FWHM peaks, could be further investigated in future work. Due to the application of the PCA to the raw *m/z* channels, a tremendous quantity of data was generated. The loading plots were therefore as complex as the mass spectra and did not help much with the interpretation, this is why we chose to not show them. The result shows that most of the variance originates from the region of the skin (spine, belly and leg) where measurements were done, which spoils attempts at species identification. However, by restricting the dataset to a single skin region, species identification is possible as mentioned above (figure 1*d*). If we limit our analysis to raw data, discrimination of the region of the skin is possible (figure 5*a*). This could be an interesting result for parchment studies since parchment's material properties may differ significantly according to the skin anatomy. Identifying the animal species is, however, of greater importance in general. Because the variance is dominated by less relevant properties, we devised a two-step PCA in which features (regions of the skin) responsible for high intra-species (intra-class) variance were first discarded. Then PCA was performed on the whole dataset but with features causing intra-class variance masked (figure 5*b*). Significant improvement of the PCA result is obtained: animal species (colours) are now discriminated, mostly along the second PC axis. Influence of the region of the skin is still noticed along the first PC axis, more specifically for spectra from the belly, which are separated from those of the two others regions (spine, leg). Nevertheless, the region on the skin is no longer responsible for the main variance, and therefore animal species identification is possible with this two-step PCA, even using a limited amount of data.

The same method was also applied to data combining both parchment sides and polarities. The corresponding results are presented in the supporting information (electronic supplementary material, figure S6). In a nutshell, the PCA applied to flesh side analysed in positive polarity (electronic supplementary material, figure S6b) gives similar results to those obtained in negative polarity (figure 5*b*). Similar separation of the PCA results was obtained along the two PC axes. Yet, species discrimination was not as efficient as for negative polarity. Such an impact on the PCA results due to polarity could be induced by the relatively low amount of spectral data, or by less specific (with reference to animal species) cations detected by ToF-SIMS. This suggests that flesh side analysis is more efficient to identify animal species. In order to explain these results, as well as to improve discrimination using grain-side data, further work is needed, either by increasing the amount of collected data or analysing the loadings of the PCs. This could allow us to determine which ionic species (molecules) are responsible for the species-related variance in the two-step PCA, but also which of them are responsible for the other variances (for instance, region on the skin).

## 3. Discussion

We have proposed a non-invasive analytical method that is able to identify organic and inorganic ink pigments on illuminated manuscripts and, at the same time, is able to identify the animal skin from which the parchment was manufactured. The method relies on ToF-SIMS, which has been proven to be safe for parchment conservation. Inked and non-inked areas of the manuscript are scanned, and mass spectrum analysis is performed in order to identify ink compositions directly. Statistical post-processing is performed on ToF-SIMS data in order to identify parchment animal species.

For the analysis of inks, identification of the suspected colour pigment is achieved by searching for characteristic mass peaks of the chemical elements and molecular fragments constitutive of that pigment. Both negative and positive polarities of ToF-SIMS spectra must be recorded in order to take account of the variable sensitivity of the technique to chemical elements and to avoid missing characteristic ions. Identification of inorganic pigment is easy because most minerals produce elemental ions—especially metals. Metal compounds are also quite stable, possess low mass and can be detected easily since the related peaks generally stand out from organic peaks. Finally, their large isotopic distribution often offers clues for their identification. The identification of organic-based pigments is more challenging, especially when the ink formulation contains a low proportion of the pigment. Typical organic pigments used in medieval inks are usually large, conjugated organic molecules, with very similar structures, while the colour they confer to the ink mixture can vary drastically with small differences in their chemical structure and therefore their mass. Moreover, detection of organic pigments and their corresponding fragments is made difficult by the presence of other parasitic organic species from the parchment itself (processed skin), from non-pigmented organic components of the ink such as fillers, or from surface contamination. Nevertheless, it is still possible to search for characteristic molecular ions and high-intensity fragments that could stand out from parasitic peaks at mid-range masses. By analysing colour and black inks on several illuminated manuscripts, we have proven that ToF-SIMS was able to identify both mineral and organic major pigments and we have validated our results with the help of two other analytical methods, XRD and ATR-FTIR. ToF-SIMS has several advantages in the context of manuscript material studies: thanks to its sensitivity (several ppm in regard to the order of the per cent for ATR-FTIR and XRD), the analysis does not require thick layers of ink on the manuscript to be successful (faded inks can be analysed), it does not alter the manuscript (dedicated experiment was done to prove its non-invasiveness), it can detect traces of compounds present in the ink that could not have been detected by XRD or ATR-FTIR (for instance, zinc and zinc oxides were detected in the blue colour of manuscript #14).

Regarding the analysis of animal skins in manuscripts, ToF-SIMS, with the help of PCA, is shown to be promising for identification of animal species as well as for identification of parchment side and origin region on the skin. Further work is needed to improve discrimination power of the technique, by collecting more data for enhanced statistical analysis or by identifying molecular fragment characteristics of each species. Building a ToF-SIMS database with parchments of known animal species could enable identification of species in old manuscripts through comparison with ToF-SIMS fingerprint of each species.

In conclusion, ToF-SIMS appears to be an interesting analytical technique for material studies of illuminated manuscripts because it potentially enables the identification of all major ink components, regardless of their nature, with minimal degradation of the sample. This technique compares favourably with proven methods, namely powder-XRD, ATR-FTIR and XRF. Indeed, powder-XRD is invasive in the configuration available to us and is efficient only for crystalline pigments. ATR-FTIR offers good potential for organic pigments but remains often less practical for most inorganic ones, as they are transparent to infrared light. XRF only offers elemental composition. In comparison, ToF-SIMS is non-invasive, can analyse both inorganic and organic pigments, and can detect many elemental and compound ions. It also offers solutions to identify parchment animal species with the help of PCA.

## 4. Methods

### 4.1. Parchment samples

Fifteen single folia of historical parchments (fourteenth–seventeenth centuries) were bought from an auction. After positive advice of a medievalist colleague regarding their poor historical value, it has been decided that they can be sacrificed for invasive analyses. For routine ToF-SIMS analysis, samples of 1 × 1 cm^2^ (size imposed by the usual sample-holder) were cut. One sample of larger size (6 × 9 cm^2^) was used for testing the non-invasiveness of ToF-SIMS for parchment, using a dedicated sample holder. As the manuscript provenance could not be tracked, they were arbitrarily labelled with numbers (from 1 to 15). Modern parchments (three fully processed skins from three relevant animal species, i.e. sheep, goat and calf) were purchased from a US manufacturer working with traditional recipes (Jesse Meyer, Pergamena). They were used as reference samples for challenging our results on animal species identification. Concretely, samples of 1 × 1 cm^2^ were cut in the parchment at different locations (spine, leg and belly regions, see electronic supplementary material, figure S1) in order to check if the anatomy of the skin had an influence on the results of our ToF-SIMS analysis. Samples were labelled according to the species (sheep, calf and goat), the position on the skin (letter and number for, respectively, column and row within a grid, close to the above-mentioned anatomical regions) and the side of the parchment (flesh and grain sides).

All parchment samples (modern and historical ones) were cleaned, following recommendations of a restorer colleague from the university library. Special care was taken for historical parchments which presented variable degrees of surface alteration and dirt. Samples were first gently rubbed with PVC-free eraser in order to remove most of the dirt. Then, whenever possible without causing degradation, especially in inked areas, parchment surfaces were further cleaned by using a solution of 3 : 1 ethanol–distilled water mixture applied with a laboratory cotton swab.

### 4.2. X-ray diffraction

For the identification of ink pigments, powder XRD was used, which required invasive sampling on sacrificial parchments. Several milligrams of ink material were scraped from the parchment with a scalpel and collected in glass capillaries (0.5 mm diameter). Samples were fixed on a rotative support. XRD analyses were performed using an STOE MP powder X-ray diffractometer using Cu K*_α_*_1_ radiation; 2*θ* XRD patterns were measured from 3° to 100° with a step of 0.015°, overnight to ensure sufficient diffraction peak intensities. Both identification and quantification of the crystalline phases were realized by comparison of the measured spectra with a database of known crystalline materials through the Highscore software.

### 4.3. Attenuated total reflection Fourier transform infrared

ATR-FTIR (Spectrum 2 FTIR spectrometer, Perkin Elmer) spectra were recorded from parchment samples in a non-invasive way, in ATR mode (diamond crystal). Spectra ranged from 450 to 4000 cm^−1^ (roughly from 2.5 to 22 µm in wavelengths), with a 4 cm^−1^ resolution, and averaging was performed on four scans. The area probed on the sample was less than 1 mm^2^ while the probed sample depth was about 1.0–1.5 µm, depending on the absorption coefficient of the material and the wavelength of the IR radiation. The pressure applied to the sample in order to ensure optimal contact with the ATR diamond crystal was kept constant for all measurements. Five spectra were recorded for each sample for repeatability purposes, at different positions. All spectra were normalized and averaged for each sample. No specific preparation of the sample was needed, except cleaning of the surface.

### 4.4. Time-of-flight secondary ion mass spectrometry

Prior to analysis, cleaned samples (1 × 1 mm^2^) were degassed in a high-vacuum (10^−8^ to 10^−7^ mbar) standalone chamber for 1 to 2 weeks in order to avoid long degassing time in the instrument. They were then transferred as quickly as possible in the pre-vacuum chamber of the ToF-SIMS instrument in order to degas overnight before transfer in the analysis chamber. They were then analysed as is, directly on the sample surface by the ToF-SIMS instrument (TOF-IV, IONTOF). Recorded spectra were processed with the Surface Lab software (developed by IONTOF). Ion beam energy was equal to 25 keV for Bi^3+^ clusters. The instrument was calibrated in mass and resolution for each polarity by using pure silicon as a reference. During parchment analysis, charge compensation was used thanks to charge neutralizer combined with argon gas injection (pressure during analysis was kept constant at 5 × 10^−7^ mbar). The instrument operated in high current bunched mode, and ion extraction bias (surface potential) was tuned for each sample. In order to get a compromise for optimal signal intensity and spectral resolution, a field of view of 500 × 500 µm^2^ was used with a lateral resolution of 128 pixels, with one mass spectrum scan per pixel each time the area was scanned. Spectra were recorded in both positive and negative polarities. Two durations of data accumulation were used. For ink identification, for which higher intensity spectra were needed, 180 s was chosen (roughly 80 accumulated scans per pixel). For animal species identification, a large quantity of data was required (i.e. for principal component analysis, high intensity is less critical than large number of spectra); spectra were recorded in 120 s (approx. 60 scans per pixel). A first peak shift (mass) calibration was performed after spectra recording.

Due to high rugosity of the samples, either in inked or non-inked parchment areas, correction was needed on measured raw data. Automatic correction algorithm from Surface Lab software was used. Basically, the time of flight variations induced by surface roughness were corrected by considering the first hundred peaks that were identified as non-overlapping ion species. Spectral resolution after that correction was two to three times higher. A second peak shift (mass) calibration was then performed once again.

For ink identification, high-intensity spectra were directly analysed thanks to the Surface Lab software (automatic peak attribution). For animal species identification, on the other hand, the large set of spectra was processed by PCA without automatic mass peak attribution. In this case, the PCA was directly applied to the raw data, i.e. *m/z* intensity channels.

### 4.5. Principal component analysis

PCA [40] is a widely used tool to extract from data with a large feature set a smaller yet comprehensive set called the principal components (PCs). PCs are sorted by decreasing order of significance. In practice, often the first two PCs are retained so that PCA results take the form of a two-dimensional scatter plot.

In ToF-SIMS spectra, the above-mentioned features are intensities at each *m/z*. Direct application of PCA on our ToF-SIMS raw data revealed that the most significant (with highest variance) features originated from parchment characteristics (regions of the skin) not related to the animal species. Therefore, PCA on raw data could not lead to species identification unless the dataset was restricted to specific cases (measurements restricted to the same region of the skin). For the general case, a two-step PCA procedure was devised. Firstly, for each species, features responsible for above-threshold intra-species (class) variance were identified and eliminated. Secondly, PCA was performed on the whole data with features causing intra-class variance masked. In some aspects, this approach is similar to only considering higher order PCs with the convenience of being continuously tunable and more explicitly focused on the classes of interest. Concretely, subsets of the data belonging to classes of interest (goat, calf, sheep) were defined. Then PCA was applied to each subset, producing loadings that measure contribution of every feature to each of the principal components. In order to mask the most impacting features for intra-class variance, a threshold was applied to loadings, and features above that threshold were ignored. The logical masks obtained for each class were assembled using the ‘or’ operator. Finally, classic PCA was applied to the reduced dataset obtained by implementing the masks.

## Data Availability

Data are available at: https://doi.org/10.5061/dryad.p8cz8w9v7 [41]. The data are provided in electronic supplementary material [42].

## References

[1] Turner NK. 2018 The materiality of medieval parchment: a response to ‘the animal turn’. Rev. Hispánica Mod. **71**, 39-67. (10.1353/rhm.2018.0007)

[2] Fiddyment S, Teasdale MD, Vnouček J, Lévêque É, Binois A, Collins MJ. 2019 So you want to do biocodicology? A field guide to the biological analysis of parchment. Herit. Sci. **7**, 1-10. (10.1186/s40494-019-0278-6)

[3] Bischoff FM. 1993 Observation sur l'emploi de différentes qualités de parchemin dans le livre médiéval. In Ancient and medieval book materials and techniques (eds M Maniaci, PF Munafò), pp. 57-94. Vatican City: Biblioteca Apostolica Vaticana.

[4] Cohen Z. 2022 *Composition analysis of writing materials in Cairo Genizah documents*, Cambridge Genizah Studies Series, vol. 15. Leiden, The Netherlands: Brill. (10.1163/9789004469358)

[5] Hahn O, Nehring G, Freisitzer R, Rabin I. 2019 A study on early European inks from St Paul in Lavanttal. Gaz. du livre médiéval **65**, 58-81. (10.3406/galim.2019.2150)

[6] Zerdoun Bat-Yehouda M. 1983 Les encres noires au Moyen âge (jusqu’à 1600). Doc. études répertoires l'Institut Rech. d'Histoire des Textes **28**, 437.

[7] Fiddyment S et al. 2015 Animal origin of 13th-century uterine vellum revealed using noninvasive peptide fingerprinting. Proc. Natl Acad. Sci. USA **112**, 15 066-15 071. (10.1073/pnas.1512264112) PMC4679014 26598667

[8] Pohl B. 2021 Documenting the everyday in medieval Europe: the social dimensions of a writing revolution, 1250–1350. Am. Hist. Rev. **126**, 370-371. (10.1093/ahr/rhab080)

[9] Clanchy MT. 2013 From memory to written record: England 1066–1307. Oxford, UK: John Wiley & Sons.

[10] Ruffini-Ronzani N. et al. 2020 *Encre, parchemin et papier à Chartres au XIVe siècle Les matériaux de l’écrit au prisme des sciences expérimentales*. Bibliothèque de l’École des chartes. See https://hal.science/hal-02292520.

[11] Glaser L, Deckers D. 2013 The basics of fast-scanning XRF element mapping for iron-gall ink palimpsests. In *Natural Sciences and Technology in Manuscript Analysis Conf, December*, vol. 7. See https://www.researchgate.net/publication/274378556.

[12] Duran A, López-Montes A, Castaing J, Espejo T. 2014 Analysis of a royal 15th century illuminated parchment using a portable XRF-XRD system and micro-invasive techniques. J. Archaeol. Sci. **45**, 52-58. (10.1016/j.jas.2014.02.011)

[13] Možir A, Gonzalez L, Kralj Cigić I, Wess TJ, Rabin I, Hahn O, Strlič M. 2012 A study of degradation of historic parchment using small-angle X-ray scattering, synchrotron-IR and multivariate data analysis. Anal. Bioanal. Chem. **402**, 1559-1566. (10.1007/s00216-011-5392-6) 21928080

[14] Odlyha M, Theodorakopoulos C, Bozec L. 2009 Fourier transform infra-red spectroscopy (ATR / FTIR) and scanning probe microscopy of parchment. e-Preservation Sci. **6**, 138-144.

[15] Graženaite E, Kiuberis J, Beganskiene A, Senvaitiene J, Kareiva A. 2014 XRD and FTIR characterisation of historical green pigments and their lead-based glazes. Chemija **25**, 199-205.

[16] Lee AS, Mahon PJ, Creagh DC. 2006 Raman analysis of iron gall inks on parchment. Vib. Spectrosc. **41**, 170-175. (10.1016/j.vibspec.2005.11.006)

[17] Čiuladienė A, Luckutė A, Kiuberis J, Kareiva A. 2018 Investigation of the chemical composition of red pigments and binding media. Chemija **29**, 243-256. (10.6001/chemija.v29i4.3840)

[18] Larsen R. 2007 Improved damage assessment of parchment. European Commission, Directorate-General for Research and Innovation.

[19] Kern MS, Pataki-Hundt A, Wouters J, Kirby DP. 2018 Accelerated ageing of parchment: investigation of a photo catalysed, low-heat approach. Restaurator **39**, 33-69.

[20] Toniolo L, D'Amato A, Saccenti R, Gulotta D, Righetti PG. 2012 The Silk Road, Marco Polo, a bible and its proteome: a detective story. J. Proteomics **75**, 3365-3373. (10.1016/j.jprot.2012.03.051) 22504796

[21] Buckley M. 2016 Species identification of bovine, ovine and porcine type 1 collagen; comparing peptide mass fingerprinting and LC-based proteomics methods. Int. J. Mol. Sci. **17**, 445. (10.3390/ijms17040445) 27023524 PMC4848901

[22] Alvarez AMF, Bouhy J, Dieu M, Charles C, Deparis O. 2019 Animal species identification in parchments by light. Sci. Rep. **9**, 1825. (10.1038/s41598-019-38492-z) 30755703 PMC6372671

[23] Ruffini-Ronzani N et al. 2021 A biocodicological analysis of the medieval library and archive from Orval Abbey, Belgium. R. Soc. Open Sci. **8**, 210210. (10.1098/rsos.210210) 34109043 PMC8170200

[24] Vilde V, Abel ML, Watts JF. 2016 A surface investigation of parchments using ToF-SIMS and PCA. Surf. Interface Anal. **48**, 393-397. (10.1002/sia.6013)

[25] Voras ZE, deGhetaldi K, Baade B, Gordon E, Gates G, Beebe TP. 2016 Comparison of oil and egg tempera paint systems using time-of-flight secondary ion mass spectrometry. Stud. Conserv. **61**, 222-235. (10.1179/2047058414Y.0000000154)

[26] Richardin P, Mazel V, Walter P, Laprévote O, Brunelle A. 2011 Identification of different copper green pigments in renaissance paintings by cluster-TOF-SIMS imaging analysis. J. Am. Soc. Mass Spectrom. **22**, 1729-1736. (10.1007/s13361-011-0171-3) 21952886

[27] Noun M, Van Elslande E, Touboul D, Glanville H, Bucklow S, Walter P, Brunelle A. 2016 High mass and spatial resolution mass spectrometry imaging of Nicolas Poussin painting cross section by cluster TOF-SIMS. J. Mass Spectrom. **51**, 1196-1210. (10.1002/jms.3885) 27615561

[28] Iorio M, Graziani V, Lins S, Ridolfi S, Branchini P, Fabbri A, Ingo G, Di Carlo G, Tortora L. 2019 Exploring manufacturing process and degradation products of gilt and painted leather. Appl. Sci. **9**, 3016. (10.3390/app9153016)

[29] Iorio M, Sodo A, Graziani V, Branchini P, Municchia AC, Ricci MA, Salvadori O, Fiorin E, Tortora L. 2021 Mapping at the nanometer scale the effects of sea-salt derived chlorine on cinnabar and lead white by using delayed image extraction in ToF-SIMS. Analyst **146**, 2392-2399. (10.1039/D0AN02350G) 33656508

[30] Zilberstein G, Zilberstein S, Maor U, Baskin E, D'Amato A, Righetti PG. 2019 *De re metallica*. Johannes Kepler and alchemy. Talanta **204**, 82-88. (10.1016/j.talanta.2019.05.094) 31357370

[31] Vnouček J, Fiddyment S, Quandt A, Rabitsch S, Collins M, Hofmann C. 2020 The parchment of the Vienna genesis: characteristics and manufacture. In The Vienna genesis, pp. 35-70. Vienna, Austria: Böhlau Verlag. (10.7767/9783205210580.35.

[32] Abdel-maksoud G, Emam H, Mahmoud N. 2020 From traditional to laser cleaning techniques of parchment manuscripts: a review. Adv. Res. Conserv. Sci. **1**, 52-76. (10.21608/arcs.2020.111216)

[33] Zhang M, Sun Y. 2013 Salvage and treatment for the archives in Beichuan earthquake. Restaur. Int. J. Preserv. Libr. Arch. Mater. **34**, 67-80.

[34] Bouvier C, Van Nuffel S, Walter P, Brunelle A. 2022 Time-of-flight secondary ion mass spectrometry imaging in cultural heritage: a focus on old paintings. J. Mass Spectrom. **57**, e4803. (10.1002/jms.4803) 34997666

[35] Atrei A, Scala A, Giamello M, Uva M, Pulselli RM, Marchettini N. 2019 Chemical composition and micro morphology of golden laminae in the wall painting ‘La Maestà’ by Simone Martini: a study by optical microscopy, XRD, FESEM-EDS and ToF-SIMS. Appl. Sci. **9**, 3452. (10.3390/app9173452)

[36] Brajković M, Barac M, Cosic D, Bogdanović Radović I, Siketić Z. 2019 Development of MeV TOF-SIMS capillary microprobe at the Ruđer Bošković Institute in Zagreb. Nucl. Instruments Methods Phys. Res. Sect. B Beam Interact. with Mater. Atoms **461**, 237-242. (10.1016/j.nimb.2019.10.006)

[37] Seki T, Nonomura T, Aoki T, Matsuo J. 2020 MeV-SIMS measurement of lithium-containing electrolyte. Nucl. Instruments Methods Phys. Res. Sect. B Beam Interact. with Mater. Atoms **479**, 229-232. (10.1016/j.nimb.2020.07.007)

[38] Mafredas T. 2010 The color palette in the Byzantine icons. Kalamata, Greece: University of the Peloponnese.

[39] Tortora L, de Notaristefani F, Ioele M. 2014 ToF-SIMS investigation of gilt and painted leather: identification of indigo, oil binder and gold varnish. Surf. Interface Anal. **46**, 807-811. (10.1002/sia.5450)

[40] Abdi H, Williams LJ. 2010 Principal component analysis. Wiley Interdiscip. Rev. Comput. Stat. **2**, 433-459. (10.1002/wics.101)

[41] Gravis D, Roy N, Ruffini-Ronzani N, Houssiau L, Felten A, Tumanov N, Deparis O. 2023 Data from: Secondary ion mass spectrometry, a powerful tool for revealing ink formulations and animal skins in medieval manuscripts. *Dryad Digital Repository*. (10.5061/dryad.p8cz8w9v7) PMC10245198 37293355

[42] Gravis D, Roy N, Ruffini-Ronzani N, Houssiau L, Felten A, Tumanov N, Deparis O. 2023 Secondary ion mass spectrometry, a powerful tool for revealing ink formulations and animal skins in medieval manuscripts. Figshare. (10.6084/m9.figshare.c.6671936) PMC10245198 37293355

